# Evaluation of forage oat cultivars for alpine saline-alkaline environments: Trade-offs between yield, quality and stability

**DOI:** 10.1371/journal.pone.0354378

**Published:** 2026-07-23

**Authors:** Yangji Jia, Haiying Zhang, Fengjuan Xue, Hao Sun, Xiaoli Wei, Xiaojian Pu, Yuanyuan Zhao, Chengti Xu

**Affiliations:** 1 College of Animal Science and Veterinary Science, Qinghai University, Xining, Qinghai Province, China; 2 Northwest Key Laboratory of Cultivated Land Conservation and Marginal Land Improvement, Ministry of Agriculture and Rural Affairs, Delingha, Qinghai Province, China; Universitat Jaume 1, SPAIN

## Abstract

Oat cultivation in the alpine saline-alkaline Qaidam Basin requires cultivars that balance yield, quality, and stability. Identifying suitable cultivars is key to sustainable forage production. Three oat cultivars (‘Qinghai 444’, ‘Qingtian No. 1', and ‘Zhengshi No. 1’) were evaluated in a two-year (2024 and 2025) field trial. We measured agronomic traits, yield, photosynthetic parameters, and forage quality. Mixed linear models, correlation analysis, and principal component analysis (PCA) were used to assess genotypic effects, environmental stability, and trait relationships. Cultivars exhibited distinct strategies. ‘Zhengshi No. 1’ achieved the highest fresh yield (3.29 and 1.96 t/ha) linked to greater stem diameter and photosynthetic rates but with high environmental sensitivity. ‘Qingtian No. 1’ provided stable, superior forage quality (highest crude protein: 11.44% and 10.33%). ‘Qinghai 444’ showed the most stable agronomic performance and highest water-use efficiency. Key trade-offs were identified, including a negative correlation between yield-related traits and forage quality (Relative Feed Value). The choice of optimal cultivar depends on primary goals: ‘Zhengshi No. 1’ for maximum biomass, ‘Qingtian No. 1’ for high quality, and ‘Qinghai 444’ for reliable performance under variable conditions. This provides an actionable framework for cultivar selection in saline-alkaline regions.

## 1. Introduction

Soil salinization is a major global environmental constraint, affecting over 1 billion hectares of land and severely limiting agricultural productivity and sustainability, particularly in arid and semi-arid regions [1,2]. With growing population pressures and climate change, the sustainable utilization of such marginal lands has become a critical global challenge for ensuring food and feed security without further ecosystem degradation [3]. This challenge is exacerbated in alpine and plateau ecosystems, where salinization often co-occurs with other abiotic stresses such as low temperatures, drought, and high radiation, creating complex multi-stress environments that are among the most challenging for crop cultivation [4].

The Qaidam Basin, situated on the northern Tibetan Plateau, is a quintessential example of such an alpine saline-alkaline environment [5]. Characterized by a harsh continental climate, saline-alkaline soils, and significant diurnal temperature variations, it presents a formidable barrier to conventional agriculture [5,6]. As a model region facing compounded stresses, it serves as a valuable case study for developing solutions applicable to other fragile ecosystems worldwide, from the Andes to the highlands of Central Asia [6]. Beyond the ecological challenge, the Basin is a vital livestock base supporting approximately 1.2 million head of livestock. However, it faces a persistent winter-spring forage deficit of up to 40%, forcing reliance on costly external supplies and undermining the sustainability of local husbandry [7]. Paradoxically, the region possesses vast tracts of underutilized saline-alkali land, estimated at 3.5 million hectares, which represents a critical potential resource for forage expansion [8]. Consequently, identifying and deploying crop species and varieties capable of resilient growth in this marginal land is not only an ecological imperative but also a socio-economic necessity for local forage security and the productive utilization of degraded areas [9].

Oat (*Avena sativa* L.) is a promising annual forage crop for stress-prone environments due to its recognized tolerance to cold, drought, and moderate salinity, alongside its high biomass yield potential and favorable nutritional quality [10,11]. These attributes make it a prime candidate for cultivation in marginal lands worldwide [11]. Specifically for the Qaidam Basin, the introduction of adapted oat varieties holds the dual promise of transforming barren saline-alkali land into productive forage fields–potentially mitigating a significant portion of the regional feed gap, while simultaneously improving soil quality through root-mediated carbon sequestration and organic matter enhancement [12]. However, substantial genotypic variation exists among oat cultivars, leading to divergent phenotypic performance, including agronomic architecture, photosynthetic efficiency, and yield–under combined stress conditions [13–15]. Critically, current cultivars used in the region are predominantly introduced from low-altitude areas and often exhibit severe yield reduction and poor adaptability under the combined alpine saline-alkaline stresses [16]. This mismatch between genetic potential and local environmental constraints is a common problem worldwide, hindering the effective exploitation of stress-tolerant crops on marginal lands [17–19]. Despite the recognized potential of oats, systematic evaluations of cultivar performance under the unique combined stresses of alpine saline-alkaline environments remain scarce. Most existing studies focus on single stresses or are conducted in more temperate saline regions. There is a clear knowledge gap regarding how different oat genotypes trade off between key agronomic objectives, such as yield, forage quality, and trait stability, when faced with the interacting constraints of cold, aridity, and soil salinity-alkalinity. This knowledge gap not only limits local agricultural development but also constrains our broader understanding of crop adaptation mechanisms in multi-stress environments, which is essential for breeding programs targeting climate-resilient agriculture globally [17].

To address this local and global gap, a two-year field study (2024 and 2025) was conducted in the Qaidam Basin to evaluate three distinct forage oat cultivars. The primary objectives were to: (1) comprehensively assess and compare their agronomic performance, photosynthetic characteristics, yield, and forage quality under these representative field conditions; and (2) identify the key traits and potential trade-offs that determine cultivar adaptability, with the aim of providing evidence-based recommendations for cultivar selection in this and similar ecoregions. The insights gained are expected to contribute not only to sustainable pastoralism in the Tibetan Plateau but also to the broader scientific framework of cultivating stress-resilient crops in marginal environments worldwide, offering a replicable methodology for genotype evaluation under complex abiotic stresses. A limitation of this study is its focus on three cultivars at a single location over two growing seasons. While this provides valuable initial insights, the findings highlight the need for future research involving a broader genetic panel across multiple sites and years to fully elucidate genotype-by-environment interactions and confirm the generalizability of the results.

## 2. Materials and Methods

### 2.1. Experimental site overview

#### 2.1.1. Experimental site and climate.

**Fig 1:**
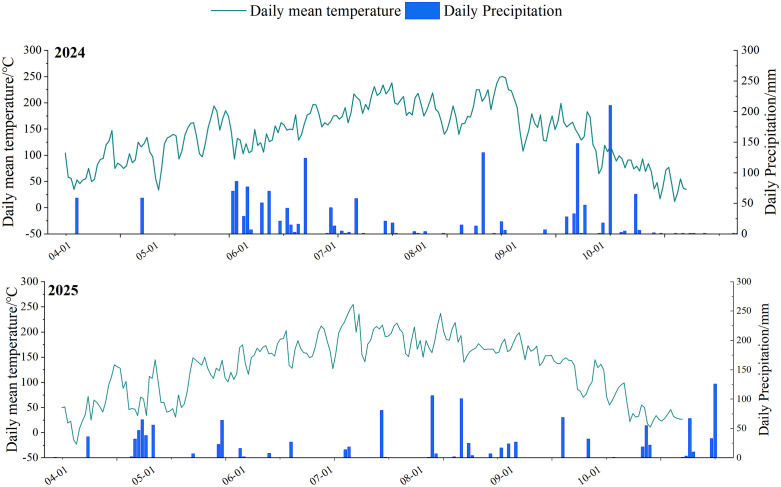
Daily precipitation and mean daily air temperature during the 2024 and 2025 oat growing seasons.

A two-year field experiment was conducted during the 2024 and 2025 growing seasons at a site in Gahai Town, Delingha City, Haixi Prefecture, Qinghai Province, China (97°22′44″ E, 37°15′13″ N, altitude 2842.3 m). The site is located on the northeastern margin of the Qaidam Basin, a region characterized by a plateau continental climate, with cold, arid conditions, strong solar radiation, and a pronounced diurnal temperature variation. The experimental field represents a typical desert saline-alkaline grassland ecosystem under managed flood irrigation. Prior to the establishment of this trial, the field was under a local common crop rotation system, predominantly alternating between forage oats (*Avena sativa* L.) and annual forage grasses (such as barley or ryegrass) or being left fallow, with forage oats being the previous crop. The daily precipitation and mean daily air temperature throughout the experimental growing seasons are shown in Fig 1.

#### 2.1.2. Soil characteristics.

To characterize the baseline conditions of the experimental site, composite soil samples were collected from the 0–30 cm depth across the entire field area before sowing in the first experimental year (2024). Physical, hydraulic, and chemical properties were analyzed using standard methods, with results summarized in Tables 1 and 2. The soil chemical profile indicated a moderately alkaline and slightly saline environment typical of the local desert saline-alkaline grassland. The field had a uniform management history before the experiment, and consistent management practices were applied across all plots during the study. Therefore, the soil properties presented here are considered representative of the initial conditions for both growing seasons.

**Table 1:** Physical and hydraulic properties of the soil (0–30 cm depth) at the experimental site.

Texture Class (USDA)	Clay (<0.002 mm) %	Silt (0.002–0.05 mm) %	Sand (0.05–2 mm) %	Bulk Density/ (g cm^-3^)	Field Capacity (–33 kPa)/ (cm^3^ cm^-3^)	Permanent Wilting Point (–1500 kPa)/ (cm^3^ cm^-3^))	Saturated Hydraulic Conductivity/ (cm day^-1^)
Sandy Loam	15.2	28.7	56.1	1.52	0.25	0.10	25

**Table 2:** Chemical properties of the soil (0-30 cm depth) at the experimental site.

Electrical Conductivity/ (dS m^-1^)	Organic Matter/ (g·kg^-1^)	Total Nitrogen/ (g·kg^-1^)	Total Phosphorus/ (g·kg^-1^)	Total Potassium/(g·kg^-1^)	Ammonium Nitrogen/(mg·kg^-1^)	Nitrate Nitrogen/(mg·kg^-1^)	Available Phosphorus/(mg·kg^-1^)	Available Potassium/(mg·kg^-1^)	Soil Salinity/ (g·kg^-1^)	Soil pH
2.40	18.13	0.83	0.44	21.72	6.39	21.87	28.08	129.61	2.40	8.46

#### 2.1.3. Applicability of findings.

The agro-ecological conditions described above are representative of the irrigated saline-alkaline desert grasslands surrounding the Qaidam Basin, particularly areas with similar soil texture (sandy loam), salinity/alkalinity levels (slightly to moderately saline-alkaline), climate (cold, arid high-altitude), and cropping systems (forage-based rotations under irrigation). Consequently, the findings related to cultivar performance, adaptation strategies, and the proposed selection framework are directly applicable to, and can be validated in, these homologous environments within the broader Qaidam Basin and similar high-altitude saline-alkaline agroecosystems on the Qinghai-Tibet Plateau.

### 2.2. Experimental design and agronomic management

#### 2.2.1. Test material and experimental design.

Three representative forage oat (*Avena sativa* L.) cultivars were selected for this study based on their documented performance and relevance to the alpine saline-alkaline environment of the Qaidam Basin:

‘Qinghai 444’ (V1): A locally improved, widely cultivated cultivar developed through systematic selection from indigenous landraces. It is well-adapted to the local climate and is recognized for its strong environmental stability and reliable performance under marginal conditions.

‘Qingtian No. 1’ (V2): A forage-specialized cultivar developed from a cross between a local sweet oat (female parent) and the introduced American cultivar ‘Mackinaw’ (male parent). It is characterized by high stem sugar content, abundant leaf biomass, and good palatability, making it suitable for high-quality forage production.

‘Zhengshi No. 1’ (V3): A high-yielding, late-maturing commercial cultivar. Its genetic background is distinct from the locally adapted materials, being derived from the cultivar ‘Heimeike’.

A two-year field experiment (2024 and 2025 growing seasons) used a Randomized Complete Block Design (RCBD) with four replications/year. To avoid spatial heterogeneity, plot assignments were re-randomized annually via Microsoft Excel’s RAND function. Each year included 12 plots (3 m × 5 m) separated by 1 m buffers. Oats were mechanically sown at a 3–5 cm depth, 15 cm row spacing, and 225 kg/ha seeding rate. All management (fertilization, irrigation, pest control) was uniform; irrigation was triggered when root-zone soil moisture fell below 60% field capacity. Agronomic traits were recorded from emergence to maturity in both years.

#### 2.2.2. Crop establishment and fertilization.

Sowing was performed mechanically on 26 May in both 2024 and 2025, with a row spacing of 15 cm, a sowing depth of 3–5 cm, and a seeding rate of 2.25 t/ha. Before sowing, a base fertilizer was applied, providing 75 kg N/ha (as urea) and 90 kg P_2_O_5_/ha (as diammonium phosphate). A top-dressing of 50 kg N/ha (as urea) was applied after each cutting event to promote regrowth.

#### 2.2.3. Irrigation and soil moisture monitoring.

An overhead sprinkler irrigation system was used to control water input. Soil volumetric water content (VWC, 0–30 cm depth) was monitored using a portable Time-Domain Reflectometry (TDR) probe (TRIME-PICO 64, IMKO, Ettlingen, Germany). Measurements were taken bi-weekly at three points in two representative plots per block. Irrigation was triggered when the average VWC fell below 60% of field capacity (i.e., < 0.15 cm^3^ cm^-3^). Weed control was primarily manual. A preventive fungicide spray (mancozeb) was applied once at the stem elongation stage. Aphid populations were monitored weekly, and insecticides were applied only when threshold levels were exceeded.

### 2.3. Measurement indicators and methods

#### 2.3.1. Photosynthetic characteristics.

Photosynthetic Characteristics: Photosynthetic characteristics were measured at the milk stage. On clear and windless days, measurements were conducted between 9:00 and 11:00 AM using a portable photosynthesis system (CI-340, CID Bio-Science, USA). For each plot, the flag leaf (the uppermost fully expanded leaf) from five randomly selected plants was used. The system’s chamber was set to maintain ambient CO₂ concentration and to track natural light levels. Net photosynthetic rate (Pn), stomatal conductance (Gs), and transpiration rate (Tr) were recorded. For each leaf, data logging began after stabilization within the chamber, and three consecutive stable readings were averaged to represent one replicate. Water use efficiency (WUE) was calculated based on the net photosynthetic rate and transpiration rate [20].


$$\begin{array}{c} \text{W}\text{UE}=\text{Pn}/\text{Tr} \end{array}$$ (1)

In the formula, WUE denotes the instantaneous water use efficiency of leaves. The unit is μmol CO_2_ / mmol H_2_O.

Measurement of Chlorophyll Content: Leaf chlorophyll content was determined using an SPAD-502 Plus handheld chlorophyll meter (Konica Minolta, Osaka, Japan). For each plot, ten plants were randomly selected, and the base, middle, and tip of the uppermost fully expanded functional leaves were measured under natural light. The average of the three measurements represented each plant’s relative chlorophyll content (SPAD value).

#### 2.3.2. Agronomic traits and yield.

When three oat varieties reached the milk stage, ten uniformly growing oat plants were randomly selected from each plot to measure absolute plant height (PH), tiller number (FN), leaf area (LA), and leaf number (LN). The stem diameter (SD) of the second basal internode was measured using a vernier caliper [21]. After assessing the agronomic traits, the border effects were excluded from each plot. A representative 1 m^2^ quadrat was then selected, and the oats were cut 5 cm above the ground. The fresh weight was immediately measured to calculate the fresh forage yield. The fresh samples were air-dried in a ventilated room until constant weight was achieved to determine the dry matter weight. The dry forage yield and fresh-to-dry ratio were calculated accordingly. The air-dried samples were ground into powder for subsequent use.

#### 2.3.3. Forage quality.

The crude protein (CP) content was determined using the Kjeldahl method, while the acid detergent fiber (ADF) and neutral detergent fiber (NDF) contents were analyzed by the Van Soest fiber analysis method. The relative feeding value (RFV) was calculated using the following formula [22].


$$\begin{array}{c} \text{D}\text{DM}\left(\%\text{DM}\right)=\text{88}.\text{9}-\text{0}.\text{779}\times \text{ADF}(\%\text{DM}) \end{array}$$ (2)

$$\begin{array}{c} \text{D}\text{MI}\left(\%\right)=\text{120}/\text{NDF}(\%\text{DM})\; \end{array}$$ (3)

$$\begin{array}{c} \text{R}\text{FV}=\text{DMI}(\%)\times \text{DDM}(\%\text{DM})/\text{1}.\text{29} \end{array}$$ (4)

In the formula: DMI (Dry Matter Intake) represents the daily voluntary dry matter consumed by an animal per unit of body weight, expressed as % BW (percentage of body weight) or kg/100 kg BW. DDM (Digestible Dry Matter): represents the portion of dry matter that can be digested and absorbed by the animal, expressed as % DM (percentage of dry matter).

#### 2.3.4. Statistical analysis.

Data were organized using Microsoft Excel 2021 (Microsoft Corp., Redmond, WA, USA). All statistical analyses, including assumption checks for normality (Shapiro-Wilk test) and homogeneity of variances (Levene’s test), were performed using SPSS 21.0 (IBM SPSS Statistics, Chicago, IL, USA). A linear mixed model was fitted to assess the effects, with oat variety as a fixed effect, trial year as a random effect, and their two-way interaction included. For factors or interactions with significant effects (*p* < 0.05), post-hoc comparisons were conducted using Duncan’s multiple range test. Principal component analysis (PCA), comprehensive evaluation, and graph generation were performed using Origin 2024 (OriginLab Corp., Northampton, MA, USA),and Spearman correlation analysis was conducted using the linkET package in R 4.5.1 (R Foundation for Statistical Computing, Vienna, Austria).

## 3. Results

### 3.1. Evaluation of agronomic traits and yield variation in different oat varieties

Mixed linear model analysis (Table 3) revealed notable differences in oat traits with respect to genotypic variation and environmental stability. Genotype (variety) had a highly significant (*p* < 0.01) effect on plant height (PH), stem diameter (SD), leaf area (LA), fresh yield (FY), stomatal conductance (Gs), transpiration rate (Tr), and water use efficiency (WUE) and a significant (*p* < 0.05) effect on leaf number (LN), crude protein (CP), and neutral detergent fiber (NDF). No significant effect was detected for tiller number (FN), dry yield (DY), or seven other traits (*p* > 0.05). Variance component analysis highlighted that environmental (year-to-year) impacts on traits differed. For FN, photosynthetic rate (Pn), and four additional traits, year-derived variance was ≤ 1% of total variation, indicating genotypic dominance and high stability. LN, LA, and four other traits showed year-induced variation of 1% to 10%, indicating good stability. PH and DY exhibited moderate stability, with yearly contributions ranging from 10% to 30%, indicating genotype-by-environment interactions. Conversely, SD, FY, and acid detergent fiber (ADF) were highly unstable, with environmental (year) effects accounting for over 70% of the total variance, indicating environmental dominance for these traits.

**Table 3:** Varietal differences and annual variation characteristics of oat traits (mixed linear model).

Traits	F-value of fixed effect (variety)	P-value	Proportion of variance explained by random effect (year)
Plant Height (PH)	19.026	<0.01	28.57%
Stem Diameter (SD)	40.817	<0.01	81.79%
Tiller Number (FN)	2.611	ns	0.00%
Leaf Number (LN)	5.641	<0.05	1.08%
Leaf Area (LA)	10.743	<0.01	4.79%
Fresh Yield (FY)	11.994	<0.01	85.38%
Dry Yield (DY)	3.190	ns	21.24%
Crude Protein (CP)	5.374	<0.05	6.73%
Neutral Detergent Fiber (NDF)	3.575	<0.05	0.35%
Acid Detergent Fiber (ADF)	1.020	ns	74.23%
Relative Feed Value (RFV)	1.641	ns	10.13%
Net Photosynthetic Rate (Pn)	0.004	ns	0.00%
Stomatal Conductance (Gs)	5.874	<0.01	0.00%
Intercellular CO₂ Concentration (Ci)	0.354	ns	0.00%
Transpiration Rate (Tr)	12.476	<0.01	5.07%
Water Use Efficiency (WUE)	13.218	<0.01	0.00%
SPAD value (SPAD)	0.255	ns	7.43%

Note: ns = not significant (*p* > 0.05); fixed effect refers to variety (different oat cultivars tested in the study) and random effect refers to year (field trials conducted across multiple growing seasons); F-value represents the F-statistic of fixed effect (variety) derived from mixed linear model analysis; proportion of variance explained denotes the percentage of total phenotypic variance attributed to the random effect (year).

**Fig 2:**
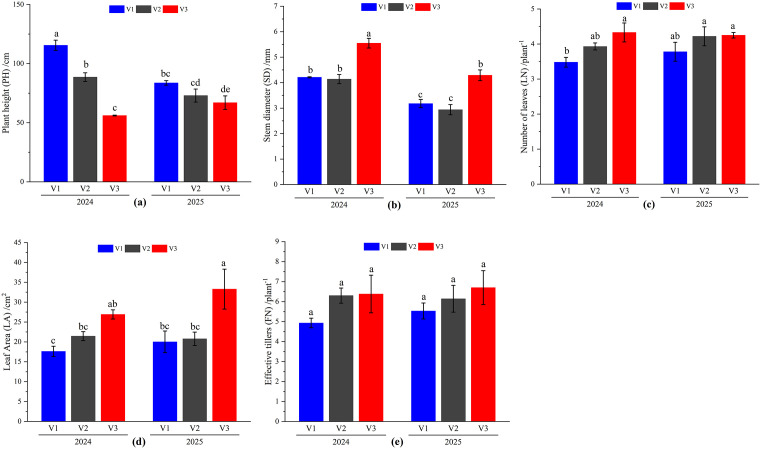
Agronomic traits of different oat varieties. Note: V1, V2, and V3 represent cultivars ‘Qinghai 444’, ‘Qingtian No. 1’, and ‘Zhengshi No. 1’, respectively. Data are presented as marginal means ± standard error (SE) estimated by the mixed linear model (*n* = 4). Within the same year, bars with different lowercase letters above indicate significant differences among cultivars, as determined by Duncan’s multiple range test (*p* < 0.05). The same abbreviations and statistical conventions are applied to all subsequent figures and tables. (a) Plant height; (b) Stem diameter; (c) Number of leaves; (d) Leaf area; (f) Effective tillers.

**Fig 3:**
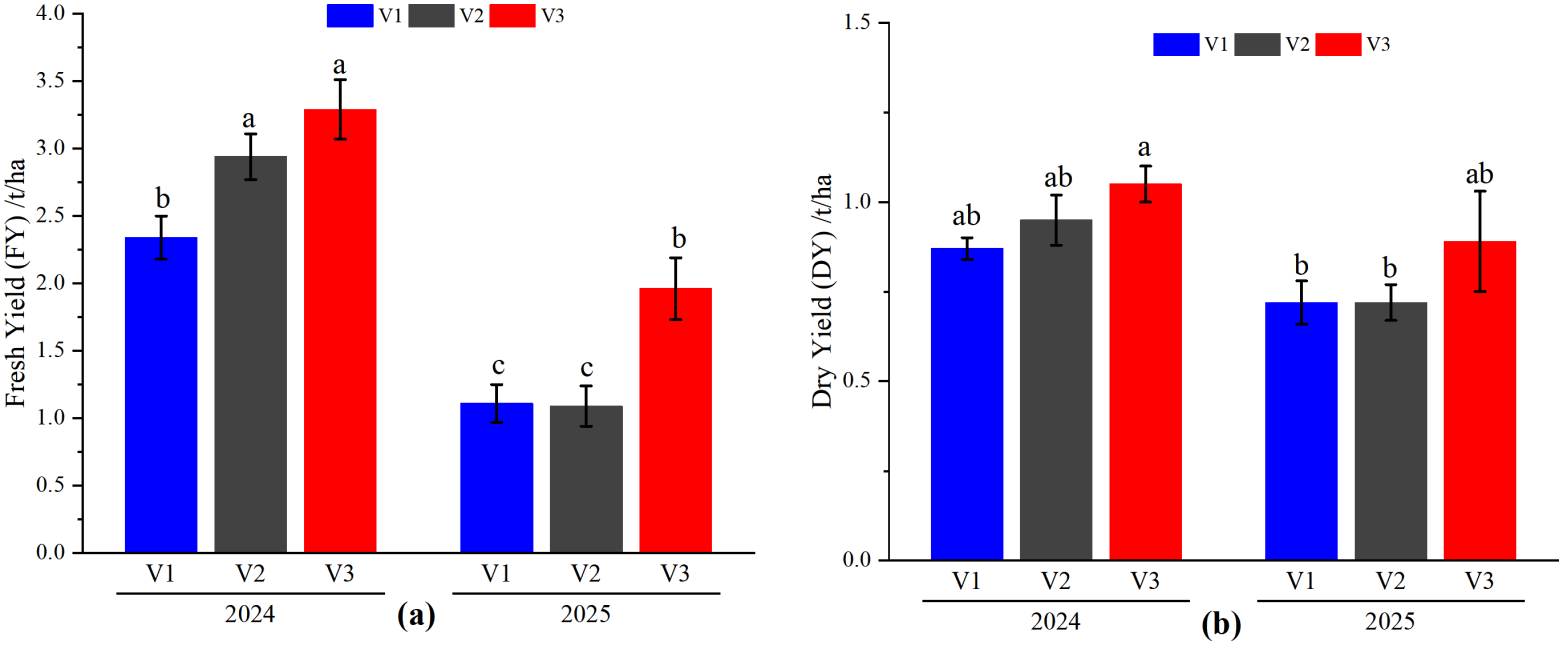
Yield of different oat varieties (2024 and 2025). Note: V1, V2, and V3 represent cultivars ‘Qinghai 444’, ‘Qingtian No. 1', and ‘Zhengshi No. 1', respectively. Data are presented as marginal means ± standard error (SE) estimated by the mixed linear model (*n* = 4). Within the same year, bars with different lowercase letters above indicate significant differences among cultivars, as determined by Duncan’s multiple range test (*p* < 0.05). The same abbreviations and statistical conventions are applied to all subsequent figures and tables. (a) Fresh yield; (b) Dry yield.

Multiple comparisons (Fig 2) further delineated with variety-specific characteristics: for PH, the order was ‘Qinghai 444’ (V1, 115.51 cm) > ‘Qingtian No. 1’ (V2, 88.59 cm) > ‘Zhengshi No. 1’ (V3, 66.99 cm), with V1 consistently the tallest and V3 the shortest across both years. In contrast, V3 performed better in SD, LA, and LN, with coefficients of variation of 3.42%/4.89% and 4.27%/15.11% across the two years for SD and LA, respectively. Although the genotypic effect on DY was not statistically significant (Fig 3), V3 showed the highest mean values for both FY and DY among the varieties tested, with FY values of 3.29 t/ha (2024) and 1.96 t/ha (2025) and DY values of 1.05 t/ha (2024) and 0.89 t/ha (2025) in the two years, indicating its potential for high yield.

### 3.2. Evaluation of nutritional quality components in different oat varieties

**Fig 4:**
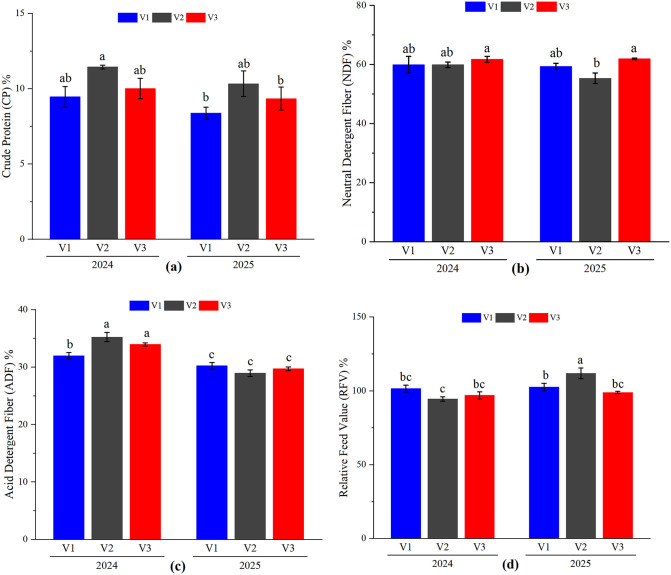
Nutritional quality of different oat varieties. Note: V1, V2, and V3 represent cultivars ‘Qinghai 444’, ‘Qingtian No. 1', and ‘Zhengshi No. 1', respectively. Data are presented as marginal means ± standard error (SE) estimated by the mixed linear model (*n* = 4). Within the same year, bars with different lowercase letters above indicate significant differences among cultivars, as determined by Duncan’s multiple range test (*p* < 0.05). The same abbreviations and statistical conventions are applied to all subsequent figures and tables. (a) Crude protein; (b) Neutral detergent fiber; (c) Acid detergent fiber; (d) Relative feed value.

Multiple comparisons for nutritional quality (Fig 4) showed that the CP content of V2 was significantly higher than that of V1 and V3 in both years, reaching 11.44% (2024) and 10.33% (2025), demonstrating a stable high-protein advantage. V1 had the lowest CP content but superior NDF content, suggesting potential for forage quality. In 2025, the relative feed value (RFV) of V2 was significantly higher than that of the other cultivars, further confirming its forage quality advantage.

### 3.3. Evaluation of photosynthetic characteristics in different oat varieties

**Fig 5:**
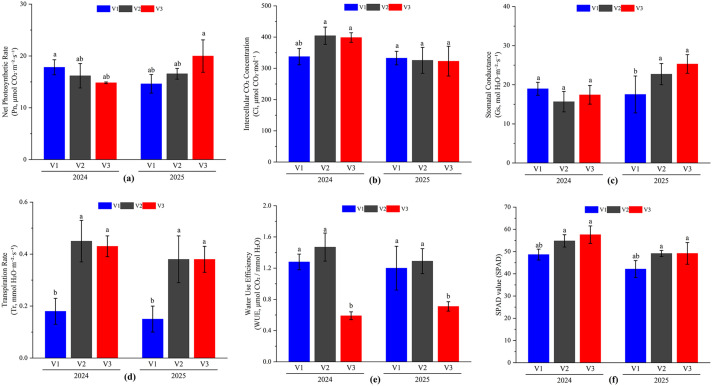
Photosynthetic Characteristics of Three Oat Varieties Over Two Consecutive Years (2024 and 2025). Note: V1, V2, and V3 represent cultivars ‘Qinghai 444’, ‘Qingtian No. 1', and ‘Zhengshi No. 1', respectively. Data are presented as marginal means ± standard error (SE) estimated by the mixed linear model (*n* = 4). Within the same year, bars with different lowercase letters above indicate significant differences among cultivars, as determined by Duncan’s multiple range test (*p* < 0.05). The same abbreviations and statistical conventions are applied to all subsequent figures and tables. (a) Net photosynthetic rate; (b) Intercellular CO₂ concentration; (c) Stomatal conductance; (d) Transpiration rate; (e) Water use efficiency; (f) SPAD value.

Photosynthetic traits, as instantaneous physiological responses, showed clear varietal differences (Fig 5). Among the tested cultivars, the net photosynthetic rate (Pn) exhibited obvious genotypic variation: V1 and V3 performed prominently in terms of Pn, reflecting their superior photosynthetic capacity. Intercellular CO₂ concentration (Ci) was higher in V2 and V3 than in V1, with V2 reaching a Ci level of 404.5 μmol·mol⁻^1^, implying a more sufficient internal CO₂ supply for carbon assimilation. Stomatal conductance (Gs), a key factor affecting gas exchange, was relatively high in V3, which provided a favorable basis for maintaining a high Pn. Transpiration rate (Tr) was consistently higher in V2 and V3 compared to V1 across the experimental period. In contrast, V1 had significantly higher water use efficiency (WUE) than V3, suggesting that this cultivar adopted an adaptive strategy to reduce water loss under saline-alkaline conditions. Regarding SPAD values, V3 was significantly higher than V1, indicating its stronger chlorophyll accumulation capacity, which may contribute to its excellent photosynthetic performance.

### 3.4. Inter-varietal trait correlation study

**Fig 6:**
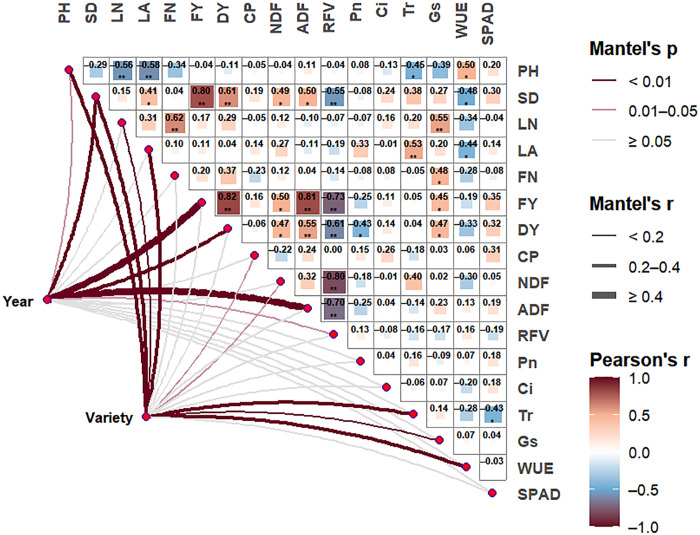
Inter-Trait Relationships in Three Oat Cultivars Over Two Consecutive Years. Note: ** indicates highly significant differences at the 0.01 level (*p* < 0.01), and * indicates significant differences at the 0.05 level (*p* < 0.05). PH、SD、LN、LA、FN、FY、DY、CP、NDF、ADF、RFV、Pn、Ci、Tr、Gs、WUE、SPAD represent plant height, stem diameter, number of leaves, leaf area, effective tillers, dry yield, fresh yield, crude protein, neutral detergent fiber, acid detergent fiber, relative feed value, net photosynthetic rate, intercellular CO₂ concentration, transpiration rate, stomatal conductance, water use efficiency, and chlorophyll content, respectively.

Mantel tests revealed significant associations between environmental/genic factors and oat trait ensembles (Fig 6). Year (environmental effect) exhibited a significant or highly significant influence on yield-related and structural traits. Specifically, it showed strong positive correlations with FY and ADF (Mantel’s r > 0.4, *p* < 0.01), and significant correlations with SD and DY (Mantel’s r = 0.2–0.4, *p* < 0.01). In contrast, cultivar (genetic effect) significantly affected a suite of traits related to plant architecture and photosynthetic physiology, including PH, SD, LN, LA, Tr, Gs, and WUE (Mantel’s r = 0.2–0.4, *p* < 0.01 for all).

Pairwise Pearson correlation analysis among the measured traits further elucidated their interrelationships (Fig 6). Of the 136 trait pairs analyzed, 29 showed significant correlations (*p* < 0.05), with 15 being highly significant (*p* < 0.01). These significant correlations included eight strong positive and seven strong negative relationships. A key finding was the strong positive correlation network among yield and growth traits: SD was positively correlated with FY (r = 0.80, *p* < 0.01) and DY (r = 0.61, *p* < 0.01), and FY was strongly correlated with DY (r = 0.82, *p* < 0.01). Conversely, a trade-off was observed between forage quality and structural/yield traits: RFV was negatively correlated with SD, FY, DY, NDF, and ADF (all *p* < 0.01). Additionally, PH was negatively correlated with LN and LA (both *p* < 0.01), suggesting a compensatory relationship between vertical growth and leaf development.

### 3.5. Comprehensive evaluation of three oat varieties based on principal component analysis

**Fig 7:**
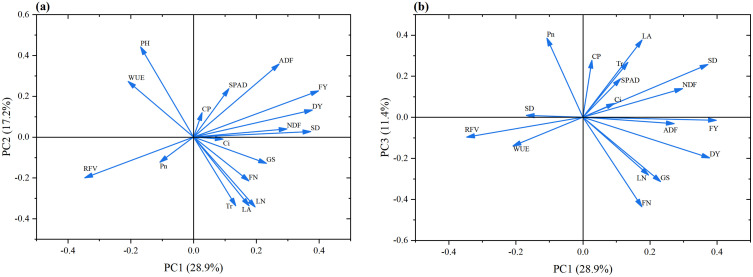
PCA Loading Plot of Various Traits in Three Oat Cultivars Across Two Years. Note: PH、SD、LN、LA、FN、FY、DY、CP、NDF、ADF、RFV、Pn、Ci、Tr、Gs、WUE、SPAD represent plant height, stem diameter, number of leaves, leaf area, effective tillers, dry hay yield, fresh grass yield, crude protein, neutral detergent fiber, acid deter-gent fiber, relative feed value, net photosynthetic rate, intercellular CO₂ concentration, transpiration rate, stomatal conductance, water use efficiency, and chlorophyll content, respectively.

A principal component analysis (PCA) was performed on 17 trait indicators of three oat varieties (Fig 7). The results showed that the first three principal components (PC1, PC2, and PC3) cumulatively explained 57.5% of the total variation. Among them, PC1 (explaining 28.9% of the variance) was primarily associated with plant growth and yield traits, exhibiting high positive loadings on FY, DY, SD, and CP, a positive correlation with fiber content indicators (NDF, ADF), and negative loadings on FN, LN, and LA. PC2 (explaining 17.2% of the variance) mainly reflected water use efficiency and quality characteristics, showing positive loadings on indicators such as CP, SPAD, ADF, FY, DY, and NDF, while exhibiting negative loadings on WUE and PH. PC3 (explaining 11.4% of the variance) was primarily related to photosynthetic physiological characteristics, with significant contributions from indicators such as Tr and Ci.

Based on the PCA loadings, comprehensive scores for each variety were calculated and ranked for both consecutive years to identify genotypes with superior overall performance (Table 4). The results showed that V3 achieved the highest comprehensive scores in both 2024 (0.566) and 2025 (0.386), significantly outperforming the other varieties, which indicates its stable and superior overall performance. V2 ranked second, with scores of 0.226 and –0.224 in the two years, respectively. In contrast, V1 recorded the lowest scores (-0.403 and -0.552), ranking third.

**Table 4:** Ranking of Three Oat Varieties Using Principal Component Analysis (PCA).

Year	Variety	F	Rank
2024	V1	−0.403	5
	V2	0.226	3
	V3	0.566	1
2025	V1	−0.552	6
	V2	−0.224	4
	V3	0.386	2

## 4. Discussion

### 4.1. Agronomic trade-offs and cultivar selection strategies

Agronomic traits serve as a critical link between genotype and phenotype, with their genetic variation directly driving yield differentiation in crops [23,24]. Our study in the alpine saline-alkaline stress environment demonstrates that key agronomic traits are not only significantly correlated with yield potential but also exhibit clear genetic variation among cultivars. Correlation analysis revealed strong positive correlations between traits such as stem diameter and yield, the mixed linear model confirmed strong genetic control over these traits, and principal component analysis further indicated that the “growth-yield” axis was the primary dimension of cultivar differentiation. These results indicate that genetic background systematically determines final yield performance by shaping distinct plant architectures, which aligns with previous research [25–27]. Cultivars V3 and V1 represent two typical resource allocation strategies. V3 achieved the highest yield by preferentially investing in biomass-related traits such as stem diameter and leaf area, constructing an efficient resource capture system. In contrast, V1 allocated resources primarily towards plant height development, forming an adaptation strategy characterized by high stability but limited yield potential. Notably, the lack of a positive correlation between plant height and yield in V1 may stem from an adaptive adjustment where excessive vertical growth becomes a limiting factor under saline-alkaline stress [28,29].

The trade-off between yield and quality is particularly pronounced under stressful conditions [30]. Classical theory posits that high yield is often accompanied by a relative decline in nutritional quality, such as protein content, partly due to the “dilution effect” of photoassimilates and priorities in resource allocation [31]. In our study, the highest-yielding cultivar V3, with its higher neutral detergent fiber (NDF) content and suboptimal crude protein (CP) and relative feed value (RFV), conforms to this pattern. However, cultivar V2 maintained the highest CP and RFV over two consecutive years while sustaining stable yield, demonstrating that yield and quality can be synergistically improved within specific genetic backgrounds. This performance may originate from more efficient nitrogen metabolism or unique resource partitioning mechanisms [32]. Therefore, this study not only validates the existence of the traditional yield-quality trade-off under stress but also, through the example of V2, indicates that this relationship is conditional, with the final phenotype determined by genotype-by-environment interactions [33,34]. V2 can serve as key germplasm for breeding high-quality and stable-yielding oat cultivars in alpine saline-alkaline regions, and the physiological and molecular mechanisms underlying its coordinated regulation of yield and quality warrant further investigation.

### 4.2. Photosynthetic and water-use strategies under stress

Photosynthesis serves as the physiological foundation and ultimate limiting factor for crop yield formation, as its efficiency directly determines dry matter accumulation and, consequently, economic yield [35]. Optimizing photosynthetic traits is a key strategy for overcoming yield bottlenecks [36]. In major cereal crops, higher photosynthetic rates are typically associated with greater yield potential, attributable to enhanced carbon assimilation capacity and efficient stomatal regulation that enables flexible responses to environmental stresses [37,38].

Under the combined stresses of cold and salinity-alkalinity in this study, significant genotypic variations in photosynthetic performance were confirmed among oat varieties, revealing distinct adaptive strategies. The V3 variety exhibited the highest photosynthetic capacity, with its elevated net photosynthetic rate (Pn) and well-maintained stomatal conductance (Gs) constituting key physiological underpinnings for its superior yield performance [39]. However, this high-yield strategy was accompanied by physiological trade-offs: the higher photosynthetic activity and transpiration rate (Tr) in V3 resulted in relatively lower water use efficiency (WUE), reflecting the common resource allocation conflict between carbon acquisition and water conservation [40,41]. In contrast, the V2 variety demonstrated greater photosynthetic stability, suggesting a more robust capacity to maintain photosynthetic machinery or more effective antioxidant protective mechanisms under fluctuating stress conditions. V1 adopted a typical “conservative” strategy characterized by higher WUE and lower Tr [42]. While this approach limits maximum photosynthetic potential, it may ensure survival and baseline yield in water-limited saline-alkaline environments by reducing transpirational water loss [43].

### 4.3. Limitations and future directions

This study provides a foundational trait-based framework, yet it is important to acknowledge its limitations. The two-year, single-site design restricts a comprehensive assessment of long-term Genotype × Environment (G × E) interactions and cultivar stability across the heterogenous conditions of the homoclime. Furthermore, while the three evaluated cultivars reveal contrasting adaptive strategies, they represent only a fraction of the available genetic diversity in oats. A notable gap is the absence of systematic data on key phenological traits, particularly days to flowering (DTF). For breeding in this constrained alpine environment, DTF is a critical adaptive trait that determines reliable crop maturation and optimal harvest scheduling, and its interaction with the yield and quality traits measured here warrants future investigation.

To address these gaps and enable the development of resilient oat varieties, future work must progress along several interconnected fronts. This includes expanding trials to multiple sites and years across the Tibetan Plateau to validate trait stability and quantify genotype-by-environment interactions, with the mandatory inclusion of days-to-flowering measurement. Concurrently, screening a broader oat germplasm collection is essential, focusing on the prioritized trait portfolio—stem diameter, water-use efficiency, and crude protein—while integrating days-to-flowering as a core selection criterion. Furthermore, leveraging the contrasting genotypes identified in this study (e.g., V2 and V3) for in-depth mechanistic research will help unravel the molecular basis of adaptation to combined stresses. Ultimately, synthesizing the knowledge gained from these expanded field evaluations, germplasm screening, and mechanistic studies will be crucial for developing integrated multi-trait selection models. These models should simultaneously optimize for yield, forage quality, abiotic stress resilience, and phenological adaptation tailored to this specific homoclime.

## 5. Conclusions

This study evaluated the performance of three oat cultivars under the unique saline-alkaline stress conditions of the region to elucidate their adaptability and inform cultivation strategies. The main findings are as follows:

Regarding ‘Zhengshi No. 1’ (V3): This cultivar demonstrated the highest fresh yield over two growing seasons, achieving 3.29 t/ha in 2024 and 1.96 t/ha in 2025, alongside superior agronomic performance and favorable photosynthetic traits. However, it also exhibited higher environmental sensitivity, with significant yield variation between years. While it showed potential for biomass production, its yield-quality trade-off—evident in lower relative feed value compared to ‘Qingtian No. 1’—suggests that its recommendation for forage use should be balanced with quality considerations.

Regarding ‘Qinghai 444’ (V1): Characterized by notable plant height and the highest water-use efficiency among the tested cultivars, ‘Qinghai 444’ displayed the most stable agronomic performance under variable conditions. Its consistent fresh yield of 2.38 t/ha (2024) and 1.58 t/ha (2025), coupled with strong physiological resilience, positions it as a reliable genetic resource for breeding programs aimed at enhancing yield stability and drought tolerance in saline-alkaline environments.

Regarding ‘Qingtian No. 1’ (V1): This cultivar excelled in forage quality, recording the highest crude protein content at 11.44% (2024) and 10.33% (2025), along with superior relative feed value. Although its fresh yield was moderate (approximately 2.41 t/ha in 2024 and 1.62 t/ha in 2025), it exhibited high environmental adaptability and physiological stability across seasons, making it an ideal candidate for ensuring consistent forage nutritional value under fluctuating climatic and edaphic conditions.

## Supporting information

S1 File Supporting data for yield, quality, and stability of forage oat cultivars in alpine saline-alkaline environments. (XLSX)

## Abbreviations

Pn: net photosynthetic rate
Ci: intercellular CO₂ concentration
Tr: transpiration rate
Gs: stomatal conductance
WUE: water use efficiency
SPAD: chlorophyll content
PH: plant height
SD: stem diameter
LN: number of leaves
FN: number of tillers
LA: leaf area
FY: fresh forage yield
DY: dry forage yield
CP: crude protein
NDF: neutral detergent fiber
ADF: acid detergent fiber
RFV: relative feed value
